# Calibration coefficients of dosimeters used in mammography for various target/filter combinations

**DOI:** 10.1120/jacmp.v16i6.5604

**Published:** 2015-11-08

**Authors:** Michiharu Sekimoto, Yoh Katoh, Tsuguhisa Katoh

**Affiliations:** ^1^ Medicine and Medical Sciences Tsukuba University Tsukuba Ibaraki Japan; ^2^ Faculty of Health Sciences Tokyo Metropolitan University Arakawa‐ku Tokyo Japan

**Keywords:** calibration coefficient, mammographic field, ionization chamber dosimeter, semiconductor dosimeter, target/filter combination

## Abstract

A wide variety of existing combinations of target and filter materials (target/filter combinations) are used in mammography equipment. The patient dose depends on the X‐ray quality that is derived from the target/filter combination, and a calibration of the dosimeter that is used in the measurement that corresponds to the specific target/filter combination is necessary. However, dosimeters in mammography are generally calibrated with reference to the X‐ray quality of Mo/Mo or W/Al combinations, and it is unclear whether or not the X‐ray quality that is derived from other target/filter combinations will affect the calibration coefficients. In this paper, the calibration coefficients of different dosimeters were evaluated for target/filter combinations. For an ionization chamber‐type dosimeter, good energy dependence was found and the effect of the target/filter combination was small. However, for a semiconductor dosimeter, a large energy dependence was found, and different calibration coefficients that depended on the target/filter combination used were required.

PACS number: 87.59.E‐

## I. INTRODUCTION

The conventional combinations of target material and filter material (target/filter combination) that have been used in mammography apparatus are mainly molybdenum/molybdenum (Mo/Mo), molybdenum/rhodium (Mo/Rh), and rhodium/rhodium (Rh/Rh). The K absorption edge of Mo or Rh filter materials is used to get a pseudomonochromatic X‐ray quality. Because the differences among the mass attenuation coefficients of the structural tissues in the human breast are small, the image contrast among these structural tissues can be enhanced in terms of the band‐pass spectrum X‐ray quality by used of a K‐edge filter.

However, when a flat‐panel detector (FPD) system is introduced into the imaging system, tungsten (W) is used as the target material, and aluminum (Al) and silver (Ag) are used as filter materials.^(1)^ The FPD offers high X‐ray detectability, a broad dynamic X‐ray detection range capability from low dose to high dose, and very good linearity between the dose and the output.^(2)^ In addition, good image processing capabilities have been obtained with the FPD, and Al filters without absorption edges are being used increasingly.^3^, ^4^ W is used as the X‐ray tube target material because it has a higher atomic number than Mo and high bremsstrahlung efficiency, allowing the high exposure rate to reduce the X‐ray tube load.^3^, ^4^, ^5^ The current target/filter combinations used in mammography apparatus are, therefore, W/Al, W/Ag, and W/Rh.^(1)^

The patient dose in mammography is evaluated as an average glandular dose (AGD). The AGD is a product of the incident air kerma and a conversion factor, g.^(6)^ Measurement of the air kerma requires an appropriate calibration coefficient. The X‐ray spectrum, the tube voltage, and the target/filter combination influence the calibration coefficients of the dosimeter. The effective energy has been used as a parameter to specify the X‐ray quality. In addition to the effective energy, the quality index (QI) parameter is also often used for specifying the beam quality, and is given by the ratio of the effective energy to the maximum photon energy.

In Japan, the calibration of the dosimeters used in mammography has generally been performed based on the X‐ray qualities of the W/Al and Mo/Mo combinations.

The available calibration facilities are those of the National Institute of Advanced Industrial Science and Technology (AIST)^7^, ^8^, ^9^ as the national standard organization and the Japan Quality Assurance Organization (JQA) as a secondary standard organization. Calibrations for combinations other than Mo/Mo and W/Al have not been performed. In this study, we evaluated the variation of the calibration coefficients of dosimeters for mammography applications with a variety of target/filter combinations.

## II. MATERIALS AND METHODS

### A. X‐ray source for calibration and filter

The X‐ray source for calibration was an industrial X‐ray generator, the ISOVOLT Titan E with an X‐ray tube, the ISOVOLT 160 with a W target, and the FA100/3 with a Mo target (GE Co., Fairfield, CT). The output windows of these X‐ray tubes were made of 1 mm thick beryllium. The coefficient of variation of the X‐ray exposure is less than 0.3%. With reference to the EUREF protocol,^(1)^ the filter thicknesses used were 0.5 mm of Al, 0.025 mm of Rh, 0.05 mm of Ag, and 0.075 mm of Ag for a W target. Mo targets were also used with filter thicknesses of 0.03 mm of Mo and 0.025 mm of Rh.

An accurate thickness, X (cm), for each filter was calculated from Eq. (1):

(1) $$X=\frac{M}{S\cdot \rho }$$

where *M* (g) is the mass measured by an electronic scale filter; the filter area *S* (cm^2^) was calculated from the formula of Heron and measurement of the four sides and the lengths of the two diagonal lines of the filter with a caliper; and ρ is the density. The densities of Al, Mo, Rh, and Ag are 2.699, 10.28, 12.41, and $10.49\;g/{\text{cm}}^{3}$, respectively. Because the AGD evaluates the air kerma passing through the compression plate, a 3 mm thick acrylic resin (polymethyl methacrylate, PMMA) layer was inserted as the corresponding filtered compression plate. It is necessary for the calibration of the dosimeter to be in the same range as the X‐ray spectrum that is actually measured, and this study was performed with the X‐ray qualities when the PMMA was included.^(10)^

### B. Reference dosimeter

**Figure 1:**
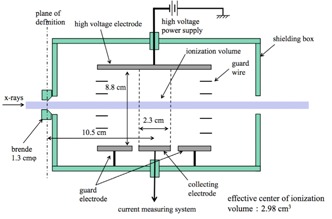
Figure of free‐air ionization chamber.

**Figure 2:**
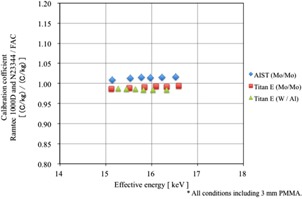
FAC was calibrated with Ramtec1000D and N23344; it has been calibrated at AIST (Mo / Mo) and JQA (W / Al). X‐axis of the graph effective energy (keV), y‐axis shows the calibration coefficient.

A free‐air chamber (FAC) was used as a reference dosimeter.^10^, ^11^ The FAC structure is shown in Fig. 1. The FAC does not require correction for photon absorption because it has small energy dependence in the effective energy region of 15 to 25 keV. The FAC would normally be calibrated at the calibration facility. However, it was difficult to take the FAC to the calibration facility. A calibration coefficient for FAC was determined using combinations of a Ramtec 1000D electrometer (Toyo Medic Co., Tokyo, Japan) and an N23344 shallow ionization chamber dosimeter (PTW Freiburg Co., Germany). The calibration coefficients of Mo/Mo at combination of Ramtec 1000D and N23344 were determined at calibration facility of the AIST and that of W/Al was determined at calibration facility of the JQA. And then, the calibration coefficients of the FAC were determined with the Titan E. The calibration coefficients of the FAC are shown in Fig. 2. The variation in the FAC calibration coefficients was within 2.5% in the effective energy range of 15 to 17 keV. From Fig. 2, the FAC calibration coefficients were 1.02.

### C. Measurement of the half‐value layer

**Figure 3:**
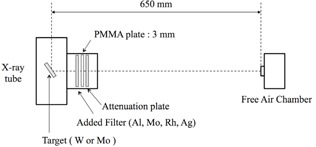
Diagram of half‐value layer measurement with a target/filter combination.

It is also necessary to calibrate a dosimeter with reference to the effective energy of the X‐rays that are used. We measured the half‐value layers to obtain the effective energy relative to the target/filter combinations. The arrangement used for the half‐value layer measurements is shown in Fig. 3. The tube voltages used were 24, 26, 28, 30, 32, and 35 kV, the tube current was 20 mA, the focus–dosimeter distance was 650 mm, and an Al attenuator was installed at a distance of 160 mm from the focus.^12^, ^13^, ^14^ The half‐value layers were calculated from the third order polynomial approximation formula determined by the attenuation curves. The attenuation coefficient (μ) was calculated from the half‐value layer, and the effective energy was calculated by use of the database of the National Institute of Standards and Technology.^(15)^

### D. Calibration of field dosimeters

**Figure 4:**
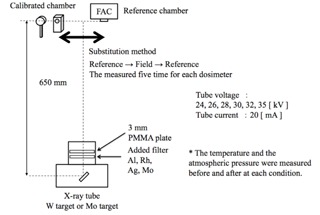
Calibration method arrangement and distance of the object to be field dosimeter with the FAC.

Field dosimeters (ionization chamber type) were investigated in combinations of a Ramtec 1000D electrometer with an N23344 chamber, an AE‐1340C electrometer with a C‐1340 chamber (Applied Engineering Co., Tokyo, Japan), and a Model 9015 electrometer with a 10X5‐6M chamber (Radcal Co., Monrovia, CA). The semiconductor type dosimeters that were studied were the Solidose 308 and the Piranha (RTI Electronics Co., Molndal, Sweden). The calibration arrangement is shown in Fig. 4. The tube voltages used were 24, 26, 28, 30, 32, and 35 kV, the tube current was 20 mA, and the focus–dosimeter distance was 650 mm. The calibration was performed with use of a substitution method, and the calibration coefficient Kc was calculated from Eq. (2):

(2) $$K_{C}=\frac{I_{S}\cdot K_{S}\cdot K_{T,P}}{I_{C}}$$

where $I_{s}$ is the indicated value of the reference dosimeter, $K_{s}$ is the calibration coefficient of the reference dosimeter, $K_{T,P}$ is the atmospheric correction coefficient, and $I_{c}$ is the indicated value of the field dosimeter. The room temperature was measured with a 0.1°C precision Hg temperature meter, and the atmospheric pressure was measured with a Fortin barometer.

## III. RESULTS

### A. Half‐value layer

The results for the half‐value layer, the effective energies, and the QIs are given in Table 1. Obtained half‐value layers were within the range described in the ACR's quality control manual.^(16)^

**Table 1:** Value (HVL, Eeff, QI) of Beam quality with a combination of target / filter and each tube voltage. All conditions including 3 mm PMMA

*Tube Voltage (kV)*		*24*	*26*	*28*	*30*	*32*	*35*
W / 0.5 mm Al	HVL (mmAl)	0.341	0.357	0.374	0.391	0.408	0.433
	Eeff (keV)	15.701	16.086	16.441	16.757	17.049	17.465
	QI	0.654	0.619	0.587	0.559	0.533	0.499
W / 0.025 mm Rh	HVL (mmAl)	0.341	0.357	0.374	0.391	0.408	0.433
	Eeff (keV)	15.274	15.529	15.776	16.016	16.252	16.587
	QI	0.636	0.597	0.563	0.534	0.508	0.474
W / 0.05 mm Ag	HVL (mmAl)	0.555	0.582	0.610	0.637	0.666	0.705
	Eeff (keV)	18.062	18.366	18.662	18.949	19.244	19.627
	QI	0.753	0.706	0.666	0.632	0.601	0.561
W / 0.075 mm Ag	HVL (mmAl)	0.661	0.685	0.709	0.733	0.759	0.792
	Eeff (keV)	19.193	19.432	19.666	19.895	20.137	20.438
	QI	0.800	0.747	0.702	0.663	0.629	0.584
Mo / 0.03 mm Mo	HVL (mmAl)	0.331	0.357	0.379	0.397	0.414	0.433
	Eeff (keV)	15.123	15.516	15.841	16.097	16.329	16.590
	QI	0.630	0.597	0.566	0.537	0.510	0.474
Mo / 0.025 mm Rh	HVL (mmAl)	0.354	0.390	0.412	0.426	0.440	0.457
	Eeff (keV)	15.479	16.002	16.306	16.495	16.679	16.898
	QI	0.645	0.615	0.582	0.550	0.521	0.483

### B. Calibration coefficient

**Figure 5:**
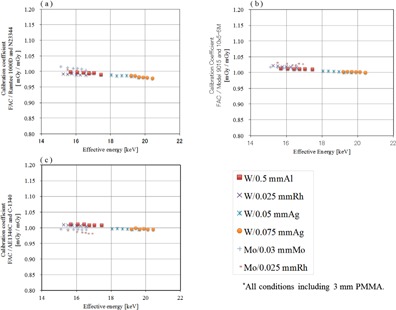
Calibration coefficients of the ionization chamber dosimeters on the basis of dosimeter free air chamber. X‐axis of the graph effective energy (keV), y‐axis shows the calibration coefficient: (a) is Ramtec 1000D and N23344; (b) the Model 9015 and 10X5‐6M; (c) is a graph showing the calibration coefficient of AE1340C and C‐1340.

**Figure 6:**
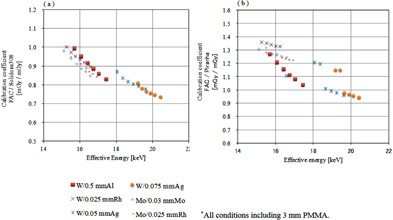
Calibration coefficients of the semiconductor type dosimeter as a reference dosimeter free air chamber. X‐axis of the graph effective energy (keV), y‐axis shows the calibration coefficient: (a) is Solidose 308, (b) is a graph showing the calibration coefficient of the Piranha.

The calibration coefficients obtained by use of the FAC are shown in Tables 2 and 3, and in Figs. 5 and 6. For the ionization chamber dosimeters, the variation with the various target/filter combinations was less than 5%. For the semiconductor dosimeters, the calibration coefficients showed large variations with the effective energy. The calibration constant obtained by calibration with reference to the X‐ray quality of the W/Ag combination was shifted away from that of the other target/filter combinations.

**Table 2:** List of the calibration coefficients of the ionization chamber dosimeter in the combination of the target / filter. All conditions including 3 mm PMMA

W / 0.5 mm Al	Ramtec 1000D and N23344	0.999	0.997	0.996	0.993	0.995	0.989
	Model 9015 and 10x5‐6M	1.013	1.012	1.011	1.011	1.011	1.010
	AE‐1340C and C‐1340	1.010	1.010	1.010	1.008	1.008	1.008
W / 0.025 mm Rh	Ramtec 1000D and N23344	0.992	0.991	0.993	0.989	0.989	0.988
	Model 9015 and 10x5‐6M	1.022	1.020	1.019	1.018	1.017	1.016
	AE‐1340C and C‐1340	1.009	1.009	1.008	1.007	1.006	1.005
W / 0.05 mm Ag	Ramtec 1000D and N23344	0.989	0.985	0.987	0.987	0.984	0.980
	Model 9015 and 10x5‐6M	1.004	1.005	1.003	1.003	1.001	1.000
	AE‐1340C and C‐1340	0.996	0.998	0.996	0.996	0.994	0.993
W / 0.075 mm Ag	Ramtec 1000D and N23344	0.986	0.986	0.982	0.982	0.980	0.978
	Model 9015 and 10x5‐6M	1.002	1.002	1.002	1.001	1.001	1.000
	AE‐1340C and C‐1340	0.996	0.998	0.995	0.996	0.995	0.994
Mo / 0.03 mm Mo	Ramtec 1000D and N23344	1.015	1.012	1.011	1.010	1.008	1.004
	Model 9015 and 10x5‐6M	1.018	1.016	1.014	1.014	1.013	1.012
	AE‐1340C and C‐1340	0.995	0.995	0.995	0.994	0.994	0.994
Mo / 0.025 mm Rh	Ramtec 1000D and N23344	1.006	1.004	1.000	0.999	0.996	0.994
	Model 9015 and 10x5‐6M	1.032	1.028	1.024	1.023	1.028	1.027
	AE‐1340C and C‐1340	0.991	0.986	0.985	0.983	0.982	0.981

**Table 3:** List of the calibration coefficients of the semiconductor detector in a combination of the target / filter. All conditions including 3 mm PMMA

W / 0.5 mm Al	Eeff (keV)	15.701	16.086	16.441	16.757	17.049	17.465
	Solidose 308	0.993	0.951	0.915	0.884	0.859	0.829
	Piranha	1.268	1.205	1.156	1.113	1.079	1.036
W / 0.025 mm Rh	Eeff (keV)	15.274	15.529	15.776	16.016	16.252	16.587
	Solidose 308	1.001	0.972	0.952	0.935	0.918	0.896
	Piranha	1.360	1.350	1.340	1.330	1.326	1.105
W / 0.05 mm Ag	Eeff (keV)	18.062	18.366	18.662	18.949	19.244	19.627
	Solidose 308	0.869	0.835	0.816	0.804	0.793	0.777
	Piranha	1.205	1.195	1.010	0.992	0.978	0.959
W / 0.075 mm Ag	Eeff (keV)	19.193	19.432	19.666	19.895	20.137	20.438
	Solidose 308	0.808	0.779	0.765	0.754	0.746	0.734
	Piranha	1.147	1.146	0.976	0.964	0.954	0.941
Mo / 0.03 mm Mo	Eeff (keV)	15.123	15.516	15.841	16.097	16.329	16.590
	Solidose 308	0.981	0.940	0.910	0.886	0.871	0.847
	Piranha	1.247	1.268	1.224	1.229	1.216	1.213
Mo / 0.025 mm Rh	Eeff (keV)	15.479	16.002	16.306	16.495	16.679	16.898
	Solidose 308	0.948	0.913	0.889	0.872	0.859	0.843
	Piranha	1.281	1.260	1.244	1.235	1.228	1.223

## IV. DISCUSSION

The calibration of a dosimeter is ideally performed with X‐rays that have the same spectrum as that of the X‐rays that are to be measured with the field dosimeter. It would be preferable, if at all possible, for the mammography apparatus to be used to generate the calibrating X‐rays. However, it is impossible for a calibration facility to prepare mammography equipment with various target/filter combinations. Furthermore, FAC of reference dosimeter is difficult to measure in mammography equipment. Therefore, industrial X‐ray generator having a W and Mo target is used as the calibration X‐ray source.

The X‐ray quality is adjusted to the X‐ray spectrum by changing the additional filter and tube voltage. The target angles vary between the X‐ray tubes of the industrial X‐ray generator and those of the mammography apparatus. However, the QIs in the present study ranged from 0.47 to 0.80, and these were within the QI range used in clinical apparatus. Therefore, calibration can also be done when an industrial X‐ray generator is well with an X‐ray spectrum that is the same as that of the mammography apparatus.

For the ionization chamber dosimeters, the calibration coefficient showed only a small variation of less than 5% with the effective energy. Wall material of mammography dosimeter is different from the dosimeter to be used in the diagnostic field. A thin film (mainly MYLAR) is used to reduce the X‐ray absorption for the incident wall. As a result, the energy dependence can be minimized. From the results, it seems that the calibration coefficient of the ionization chamber dosimeter was not affected seriously by beam quality. Therefore, a current traceability system can be used without creating problems for an ionization chamber dosimeter.

**Figure 7:**
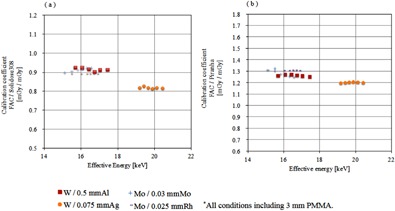
Calibration coefficients of semiconductor dosimeter plus a correction factor. X‐axis of the graph effective energy (keV), y‐axis shows the calibration coefficient: (a) is Solidose 308, (b) is a graph showing the calibration coefficient of the Piranha.

In contrast, the energy characteristics of the semiconductor dosimeter were poor, and the differences in the calibration constants relative to the effective energy were large. Si is mainly used in semiconductor dosimeters, and a depletion layer formed by a p‐n junction acts as the radiation sensing layer. Because of the structure of the semiconductor dosimeter, the X‐ray absorption by the ${\text{SiO}}_{2}$ layer of the outer layer and by a nonsensing layer can be high, and largely depends on the energy of the low‐energy region X‐rays.^(7)^ Therefore, calibration is necessary based on the effective energy of the X‐rays that are actually measured. Semiconductor dosimeters have correction factors relative to the target/filter combinations used that are proposed by their manufacturers.^(17)^ It was confirmed that the change in the calibration constants caused by the energy dependency was reduced. Piranha's correction constants, in which the correction factor was multiplied, are shown in Fig. 7. However, it is difficult to perform the appropriate calibration in Japan because the correction factor cannot be changed easily.

**Figure 8:**
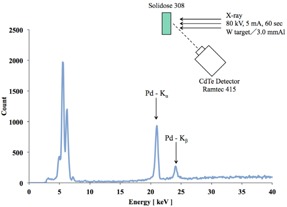
Scattered X‐ray spectra irradiated from the Solidose 308 showed the results of the measurement in CdTe detector.

The calibration coefficient of the semiconductor dosimeter under X‐rays of the W/Ag target/filter combination was higher than those produced by other target/filter combinations. The characteristic of the Pd that is built into the Si detector is believed to be the cause of this behavior. The atomic numbers and the K absorption edge energies of Rh, Pd, and Ag are 45, 46, and 47, and 23.33, 24.35, and 25.51 keV, respectively. The K absorption edge energy of Ag is higher than that of Pd. The existence of Pd in the probes of the semiconductor dosimeters was confirmed by fluorescent X‐ray analysis. The semiconductor dosimeter probes were irradiated at 65 cm from the W target with 3 mm Al filtration at 80 kV and 5 mA. The fluorescence was detected by a CdTe semiconductor spectrometer (Ramtec 415; Toyo Medic) placed 10 cm from the probe at an angle of 45°. The $K_{\alpha }$ and $K_{\beta }$ radiation of Pd was clearly observed, as shown in Fig. 8. The Pd filter thickness was estimated from the spectra of the calibration beams and the measured responses based on Burin's cavity theory.^(18)^ The spectra of the calibration X‐rays was measured with the Ramtec 415. The semiconductor detector reading was assumed to be proportional to the Si collision kerma $K_{\text{Si}}$ for the Pd‐filtered spectrum. The detector response was assumed to be proportional to the ratio of $K_{\text{Si}}$ to the air collision kerma for the same spectrum. By assuming a Pd filter thickness of 50 μm, the measured energy characteristics specific to the W/Ag X‐rays were explained well. (The details of the Pd filter will be discussed elsewhere; they are beyond the scope of this paper.)

## V. CONCLUSIONS

In this study, the variations in the calibration coefficients of dosimeters used in mammography relative to the target/filter combination used were investigated. For ionization chamber dosimeters, good energy dependencies were observed, and the effect of the target/filter combination was minimal. However, for the semiconductor dosimeters, large energy dependencies were observed and different calibration coefficients depended on the target/filter combinations were needed. In the case of using the semiconductor dosimeter, it is important to get the calibration coefficients corresponding to the X‐ray energy to be measured.
